# HEXIM1 Peptide Exhibits Antimicrobial Activity Against Antibiotic Resistant Bacteria Through Guidance of Cell Penetrating Peptide

**DOI:** 10.3389/fmicb.2019.00203

**Published:** 2019-02-08

**Authors:** Pooi Leng Ho, Han Kee Ong, Jeanette Teo, Dave Siak-Wei Ow, Sheng-Hao Chao

**Affiliations:** ^1^Microbial Cells, Bioprocessing Technology Institute, Agency for Science, Technology and Research, Singapore, Singapore; ^2^Expression Engineering Groups, Bioprocessing Technology Institute, Agency for Science, Technology and Research, Singapore, Singapore; ^3^Department of Laboratory Medicine, National University Hospital, Singapore, Singapore; ^4^Department of Microbiology and Immunology, National University of Singapore, Singapore, Singapore

**Keywords:** antimicrobial peptide, HEXIM1, antibiotic resistant bacteria, carbapenem-resistant, translation

## Abstract

The emergence of antibiotic resistant bacteria is one of the biggest threats to human health worldwide. In 2017, World Health Organization listed the world’s most dangerous antibiotic-resistant bacteria or “superbugs,” such as carbapenem-resistant *Pseudomonas aeruginosa* and *Escherichia coli*, indicating the highest priority needs for new antibiotics. The possibility that such infectious diseases may soon be untreatable, due to decreased antibiotic efficacy, creates an urgent need for novel and alternative antimicrobials. Antimicrobial peptides are naturally occurring small molecules found in the innate immunity of mammals, plants and bacteria, and are potentially therapeutic candidates against drug-resistant bacteria. In this study, we examine the antimicrobial activities of the cytotoxic peptides derived from the basic region (BR) of the human hexamethylene bisacetamide-inducible protein 1 (HEXIM1). We found that, when fused with a cell penetrating peptide, the HEXIM1 BR peptide and its derivative, BR-RRR12, exhibited inhibitory activities against selected “superbugs.” Negligible effects on the viability of human keratinocyte cell line were observed when the bactericidal dosages of HEXIM1 BR peptides were used. Different killing kinetics were observed between the membrane permeabilizing antimicrobial peptides and HEXIM1 BR peptides, suggesting that a different antimicrobial mechanism might be utilized by the HEXIM1 BR peptides. Using an *in vitro* translation system based on *E. coli* lysates, we found that HEXIM1 BR peptides blocked bacterial translation. Taken together, we identify the HEXIM1 BR peptide as a novel antimicrobial peptide with potent inhibitory activity against antibiotic-resistant “superbugs.”

## Introduction

The emergence of antibiotic resistant bacteria worldwide has become a serious concern and this has been identified as one of the biggest current threats to human health by the World Health Organization [WHO] (2014). In 2017, for the first time, WHO listed twelve families of antibiotic-resistant bacteria or “superbugs,” which posed huge threat to human health as the clinicians might be running out of treatment options (World Health Organization [WHO], 2017). The rapid rise and spread of antibiotic resistance has been attributed to the overuse and misuse of antibiotics, as well as low discovery rate of new antibiotics (Boucher et al., 2009; Ho et al., 2010; Ventola, 2015). The possibility that infectious diseases may soon be untreatable, due to decreased antibiotic efficacy, creates an urgent need to search for alternatives which are capable of killing resistant bacteria as well as having low likelihood of developing resistance against them (Hancock and Sahl, 2006; Fischbach and Walsh, 2009; Hein-Kristensen et al., 2013; Heulot et al., 2017).

Antimicrobial peptides are naturally occurring small molecules involved in the innate immunity of many organisms, such as humans, insects, plants and microorganisms. They play an important role in the first line of defense against invading pathogenic organisms (Guilhelmelli et al., 2013; Scocchi et al., 2016). Antimicrobial peptides generally have short length (9–100 residues), are cationic and amphipathic due to high proportion of arginine, lysine and hydrophobic residues (Fan et al., 2011; Scocchi et al., 2016). The killing mechanism of most antimicrobial peptides is the disruption of membrane organization by depolarization through hydrophobic and electrostatic interaction between the positively charged peptides and negatively charged lipids on bacterial cell membrane (Shai, 2002; Brogden, 2005; Guilhelmelli et al., 2013; Scocchi et al., 2016). Other mechanisms affecting microbial viability include the inhibition of DNA, RNA, protein and cell wall synthesis by targeting the essential intracellular factors (Scott and Hancock, 2000; Cudic and Otvos, 2002; Guilhelmelli et al., 2013; Scocchi et al., 2016). Development of resistance to antimicrobial peptides is considered unlikely as microbes will require significant alteration to their gene sequences, membrane structure and lipid composition to evade the peptides (Zasloff, 2002; Hein-Kristensen et al., 2013; Ravensdale et al., 2016). For this reason, coupled with its broad-spectrum activity and low host toxicity, antimicrobial peptides have gained more attention for development as therapeutic candidate against drug-resistant bacteria (Hancock, 2001).

We have previously identified a cytotoxic peptide derived from the basic region (BR) of hexamethylene bisacetamide-inducible protein 1 (HEXIM1) (Neo et al., 2016). HEXIM1 is a multi-functional protein in mammals and best known as the inhibitor of positive transcription elongation factor b (P-TEFb), which is a master regulator of RNA polymerase II transcription (Chao et al., 2000; Chao and Price, 2001; Wittmann et al., 2003; Shimizu et al., 2005; Gurumurthy et al., 2008; Lau et al., 2009; Ding et al., 2013; Ketchart et al., 2013; Morchikh et al., 2017). The BR of HEXIM1 is required to regulate the activity of P-TEFb (Michels et al., 2003; Yik et al., 2003). In addition, the BR contains a stretch of basic residues and exhibits sequence similarity to C-terminal region of p53, which can be ubiquitinated by human double minute-2 protein (HDM2) (Michels et al., 2003; Yik et al., 2003; Lau et al., 2009). As the C-terminal region of p53 had been shown to activate wild-type and mutant p53, we reasoned that the BR peptide might exhibit similar anti-cancer activity (Selivanova et al., 1997). We found that the BR peptide did not cause any non-specific cytotoxicity as it could not enter cells by itself. However, when directed by specific targeting peptides, HEXIM1 BR peptides exhibited potent toxicity against a couple of cancer cell lines (Neo et al., 2016). Unexpectedly, we found that the cell killing mechanism induced by the HEXIM1 BR peptide did not depend on p53 status. Once inside the cells, the BR peptide was mainly located in nucleoli and caused a decrease in the protein level of NPM, a nucleolar protein (Neo et al., 2016). As nucleolus is the site of ribosome biogenesis and NPM also plays a role in protein translation, the HEXIM1 BR peptide may have an impact on the machinery of protein translation.

Recent studies have shown that a subset of anticancer peptides is also cytotoxic against microbial cells (Hoskin and Ramamoorthy, 2008; Gaspar et al., 2013; Aghazadeh et al., 2018). It is possible that such peptides attain their antimicrobial activities by targeting molecular or cellular pathways that shares similar characteristics and are evolutionally conserved between bacterial and mammalian cells (Chu et al., 2015; Deslouches and Di, 2017). Since the HEXIM1 BR peptide consists of several lysine and arginine residues and is highly positively charged (amino acid sequence of the BR peptide, QLGKKKHRRRPSKKKRHW), we hypothesized that the BR peptide could also have antimicrobial properties. In this study, we demonstrate the antimicrobial activity of the HEXIM1 BR peptide against the several critical/high priority “superbugs” listed by WHO and further investigate the antimicrobial mechanism mediated by the BR peptide.

## Materials and Methods

### Bacterial Strains and Mammalian Cell Lines

Bacterial strains used in this study are listed in Table 1. Bacterial strains were either purchased from American Type Culture Collection (ATCC, United States) or provided by National University Hospital, Singapore. Prior to assays, all bacteria, except for *Enterococcus faecalis*, were grown overnight to stationary phase at 37°C in Luria Bertani (LB) broth (Novagen, Germany). The overnight cultures were 10-fold diluted in fresh cation-adjusted Mueller Hinton II broth (MHBII) (Becton Dickinson, United States) and grown for additional few hours at 37°C to obtain mid-log phase culture. *E. faecalis* strains were grown in M17 broth (Becton Dickenson, United States) for both overnight and mid-log phase culturing. Human keratinocyte cell line, HaCaT, was purchased from Creative Bioarray (United States) and cultured in Dulbecco’s Modified Eagle’s medium with 4.5 g/L glucose, 2 mM L-glutamine, and 10% fetal bovine serum.

**Table 1:** Bacterial strains used in this study.

Bacterial strains	Characteristics	Source
**Gram-Negative bacteria**		
*Escherichia coli* 25922	Sensitive control strain for susceptibility testing	ATCC^a^
*Escherichia coli* BAA2523	Resistant control strain. Carbapenem-resistant.	ATCC
*Pseudomonas aeruginosa* 27853	Sensitive control strain for susceptibility testing	ATCC
*P. aeruginosa* 544	Resistant clinical isolate. Ciprofloxacin, gentamicin and carbapenem-resistant	Clinical
**Gram-Positive bacteria**		
*Staphylococcus aureus* 29213	Sensitive control strain for susceptibility testing	ATCC
*S. aureus* 43300	Methicillin-resistant *S. aureus* (MRSA) control strain. Cefoxitin and methicillin resistant	Clinical
*Enterococcus faecalis* SEN	Sensitive clinical isolate	Clinical
*E. faecalis* VRE	Resistant clinical isolate. Vancomycin resistant	Clinical

*^a^American Type Culture center.*

### Peptides

Peptides used in this study and their amino acid sequences are listed in Table 2. All peptides were synthesized by Axil Scientific (Singapore) with >98% purity. The peptides were solubilized in molecular grade water to yield stock solution of 10 mM, aliquoted into smaller volumes, and stored at −20°C until usage.

**Table 2:** Peptides used in this study.

Name	Amino acid sequence	Reference
BR	QLGKKKHRRRPSKKKRHW	Neo et al., 2016
BR-RRR12	QLG**RRR**HRRRPS**RRR**RHW	Neo et al., 2016
Pen-BR	RQIKIWFQNRRWGGQLGKKKHRRRPSKKKRHW	This study
Pen-RRR	RQIKIWFQNRRWGGQLG**RRR**HRRRPS**RRR**RHW	This study
Cecropin P1 (CECP1)	SWLSKTAKKLENSAKKRISEGIAIAIQGGPR	Lee et al., 1989
Cap11-1-18m^2^ (CapM2)	KLRKLFRKLLKLIRKLLR	Okuda et al., 2009

### Antimicrobial Activity

Minimum inhibition concentrations (MICs) were determined by using broth microdilution method in a 96-well microtiter plate according to guidelines of the Clinical and Laboratory Standards Institute (CLSI, 2012). Briefly, mid-log phase cultures were diluted with MHBII and added to wells containing serially diluted peptides to give final cell concentration of 5 × 10^5^ cells ml^−1^. Peptides were prepared in twofold serial dilution series to give a final concentration range from 2 to 128 μM. Cecropin P1 (CECP1) and Cap11-1-18m^2^ (CapM2) treated cells were used as positive controls, while untreated cells were used as a negative control. The MIC values were defined as the lowest concentrations at which no visible growth turbidity was observed in the wells after incubation at 37°C for 20 h without shaking.

To further assess the potency of peptides, wells with no visible growth from MIC tests were subsequently plated on LB agar plates and incubated overnight at 37°C. Minimum bactericidal concentration (MBC) was determined as lowest concentration that showed no colony growth on agar plates after overnight incubation. Both MIC and MBC assays were conducted on three to five separate occasions and if results were within one doubling dilutions of each other, the highest reading was recorded for analysis.

### Bactericidal Kinetics

Bacterial killing kinetics was evaluated based on colony counts obtained after different exposure time of bacterial cells to the peptides. The assay was performed on *Escherichia coli* ATCC 25922 by incubating 5 × 10^5^ cells ml^−1^ with peptides at 2× MBC concentrations, as determined above. The peptide-bacterial mixture was incubated at room temperature for 4 h, and cell viability was determined at every 30 min time interval. Initial cell count was conducted 5 min prior to addition of peptides to determine the initial cell concentration. Aliquots of the mixture were withdrawn and plated on LB agar either in neat or diluted concentration depending on the time interval taken. The resultant colonies were counted after an overnight incubation of the plates at 37°C. Three independent tests were conducted and killing rate was plotted as log CFU ml^−1^ against time.

### *In vitro* Protein Synthesis Assay

The method to study the effect of peptide treatment on *in vitro* expression of protein was adapted from previous study (Taniguchi et al., 2016). RTS^TM^ 100 *E. coli* HY Kit (biotechrabbit) was used as a cell-free rapid translation system (RTS) to express green fluorescent protein (GFP) with or without peptide treatment. Streptomycin (Sigma-Aldrich), an antibiotic functioning as an inhibitor of bacterial translation, was used as a positive control. Reaction mixture was prepared as described in the product manual. For the negative control, the remaining 10 μl was topped up with nuclease-free water while for the other reactions, the 10 μl consists of 5 μg GFP mRNA and either nuclease-free water or the indicated treatment (100 μM Pen, Pen-BR, Pen-RRR, CapM2 or 10 μM Streptomycin). Reaction was incubated at 30°C for 6 h before being analyzed with Western blot for the protein level of GFP. To obtain GFP mRNA, control GFP expression vector was first linearized using ApaLI restriction enzyme (New England Biolabs) and then separated via agarose gel electrophoresis. Fragment containing the linearized GFP expression vector was retrieved using FavorPrep GEL Purification Kit (FAVORGEN Biotech Corp.) and subsequently used as the template for MEGAscript^TM^ T7 Transcription Kit (Thermo Fisher Scientific) to generate GFP mRNA.

### Western Blot and Coomassie Blue Staining

To study the GFP protein level in the RTS following peptide treatment, Western blot was carried out using 5 μl of the incubated reaction. SDS–PAGE was performed using NuPAGE^TM^ 4–12% Bis-Tris Protein Gels (Thermo Fisher Scientific), followed by a transfer step using iBlot 2 Dry Blotting System (Thermo Fisher Scientific). Blocking was achieved by incubating with 5% skim milk. Primary antibody used was anti-GFP mouse monoclonal antibody (sc-9996, Santa Cruz Biotechnology) while anti-mouse antibody conjugated with horseradish peroxidase was coupled with SuperSignal^TM^ West Pico PLUS Chemiluminescent Substrate (Thermo Fisher Scientific) for signal detection. Gel after the transfer step was fixed in 10% acetic acid/40% methanol for 10 min and washed with water for 20 min before staining with Bio-Safe^TM^ Coomassie Stain (Bio-Rad). Images for Western blot and Coomassie blue staining were taken using ChemiDoc^TM^ Touch Imaging System (Bio-Rad).

### Viability Assay for Mammalian Cells

HaCat cells (1 × 10^5^ cells mL^−1^) were seeded in a 96-well clear-bottomed white plate (Corning) in Dulbecco’s Modified Eagle’s medium with 4.5 g/L glucose, 2 mM L-glutamine, and 10% fetal bovine serum and incubated overnight at 37°C. On the following day, cells were treated with the indicated peptides at serial-diluted concentrations and incubated overnight. CellTiter-Glo reagent (Promega) was utilized to determine the cell viability based on the manufacturer’s instructions.

## Results

### Antimicrobial Activity of HEXIM1 BR Peptides

To determine whether the HEXIM1 BR peptides exhibit antimicrobial effect, apart from its anticancer activity (Neo et al., 2016), the BR peptides were tested against a panel of antibiotic sensitive and resistant bacteria, including Gram-negative *Escherichia coli* and *Pseudomonas aeruginosa* as well as Gram-positive *Staphylococcus aureus* and *E. faecalis* (Table 1). Four antibiotic-resistant “priority pathogens” or “superbugs” listed by WHO were examined in our study. These are the carbapenem-resistant *Escherichia coli* BAA2523 and *P. aeruginosa* 544 (both are WHO’s “priority 1: critical pathogens”), methicillin resistant *S. aureus* 43300 and Vancomycin resistant *E. faecalis* VRE (both are WHO’s “priority 2: high pathogens”) (World Health Organization [WHO], 2017). The BR mutant peptide, BR-RRR12, in which six lysine residues are mutated to arginine, was also utilized in this study (Table 2). The values of MICs and MBCs obtained from the antimicrobial study are summarized in Table 3. The BR peptide showed no signs of antibacterial activity within the concentrations tested, except for *S. aureus* strains which were inhibited, but not killed, at high concentration of 128 μM (Table 3). The BR-RRR12 peptide exhibited better inhibitory activity against a few antibiotic sensitive (*E. coli* ATCC 25922, *S. aureus* 29213, and *E. faecalis* Sensitive) and antibiotic resistant (*S. aureus* 43300 and *E. faecalis* VRE) bacteria with MICs between 16 and 64 μM. However, it was unable to inhibit any of the *P. aeruginosa* strains and antibiotic resistant *E. coli* BAA2523 (Table 3). In terms of bactericidal activity, BR-RRR12 peptide was only able to elicit toxic effect against *E. coli* 25922 with MBC value at 64 μM (Table 3).

**Table 3:** Antimicrobial and bactericidal spectrum of peptides.

	BR		BR-RRR12		Pen-BR		Pen-RRR		CapM2		CECP1	
	MIC (μM)	MBC (μM)	MIC (μM)	MBC (μM)	MIC (μM)	MBC (μM)	MIC (μM)	MBC (μM)	MIC (μM)	MBC (μM)	MIC (μM)	MBC (μM)
**Antibiotic-susceptible bacteria**												
*E. coli 25922*	>128	>128	64	64	8	16	8	16	16	32	4	4
*P. aeruginosa* 27853	>128	>128	>128	>128	8	16	8	8	16	32	16	32
*S. aureus* 29213	128	>128	32	>128	8	16	8	16	16	32	>128	>128
*E. faecalis* SEN	>128	>128	32	>128	8	16	8	16	16	16	>128	>128
**Antibiotic-resistant bacteria**												
*E. coli* BAA2523	>128	>128	>128	>128	16	32	16	16	32	64	64	64
*P. aeruginosa* 544	>128	>128	128	>128	8	8	8	8	16	32	16	32
*S. aureus* 43300	128	>128	16	128	4	16	8	8	16	16	>128	>128
*E. faecalis* VRE	>128	>128	16	>128	4	16	8	8	16	16	>128	>128

*Bacteria (5 × 10^6^ CFU ml^−1^) were incubated with twofold dilution series starting from 128 μM peptide stock solution. MIC results are determined after 20 h incubation at 37°C. MBC results are determined after plating the overnight peptide-culture on LB agar and further incubated for another 24 h at 37°C. Values in the table are the lowest concentration value that shows no visible growth (MIC) or no colony growth on LB agar plates after overnight incubation (MBC). Results that show no inhibition or bactericidal activity in the tested concentration range are indicated as >128 μM.*

In our previous study, we found that the BR peptide could not enter mammalian cells by itself and thus failed to cause any detectable cytotoxic effects. Limited toxicity against mammalian cells was observed when cells were treated with BR-RRR12 peptide at a high concentration (i.e., 100 μM) (Neo et al., 2016). Sequence similarity between BR-RRR12 (QLGRRRHRRRPSRRRRHW) peptide and a cell penetrating peptide (Arg)_9_ (RRRRRRRRR) suggested a weak cell penetrating ability of BR-RRR12 (Herce et al., 2009), resulting in non-specific cytotoxicity.

To overcome the poor cell penetrating ability of HEXIM1 peptides, we fused both HEXIM1 BR and BR-RRR12 peptides with a cell penetrating peptide, Pen, to generate Pen-BR and Pen-RRR peptides (Supplementary Figure S1). Pen peptide was derived from the original Penetratin cell penetrating peptide and was used in an earlier study as a cell penetrating peptide for *E. coli* and *S. aureus* (Schmidt et al., 2014). Both Pen-BR and Pen-RRR peptides showed improved and potent bacterial inhibitory (MIC values between 4 and 16 μM) and killing activities (MBC values between 8 and 32 μM). Pen-RRR exhibited similar or stronger bactericidal activity than Pen-BR against antibiotic resistant bacteria as indicated by the lower MBC values (Table 3). No toxic effects were observed in the bacteria incubated with the penetrating peptide, Pen (up to 128 μM; data not shown). As HEXIM1 BR peptides require the guidance provided by a penetrating peptide to elicit antibacterial activity, it suggests that membrane disruption may not be the main antibacterial mechanism of the BR peptides.

We next examined the effects of two known antimicrobial peptides, Cecropin P1 (CECP1) and Cap11-1-18m^2^ (CapM2) for comparison with our HEXIM1 BR peptides. In general, CapM2 showed similar or stronger inhibitory and killing activities than CECP1 against most bacteria examined (except for *E. coli* 25922) (Table 3). However, when compared to the HEXIM1 BR peptides, both CapM2 and CECP1 were less effective than Pen-BR and Pen-RRR with regards to the antibacterial activities (Table 3). Among the antimicrobial peptides studied here, Pen-RRR showed the best bactericidal effects against the antibiotic-resistant bacteria (Table 3, MBC values).

### Kinetic Analysis of HEXIM1 BR Peptides

**FIGURE 1:**
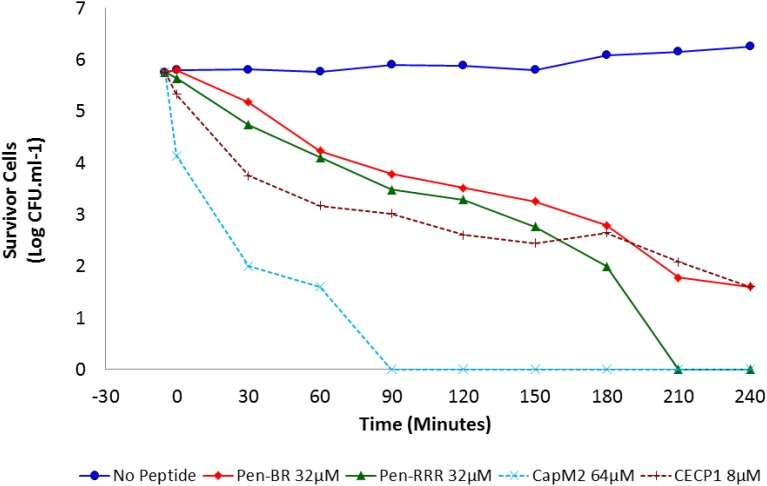
Bactericidal kinetics of Pen-BR and Pen-RRR. *E. coli* 25922 cells were incubated with peptides at 2× MBC values (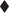
32 μM Pen-BR; 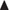
32 μM Pen-RRR; ×64 μM CapM2; +8 μM CECP1) for 4 h. Cell counts were conducted 5 min before incubation with peptides and every 30 min interval after addition of peptides.

Bacterial killing kinetics for Pen-BR, Pen-RRR and control peptides (CapM2 and CECP1) were determined at 2× MBC on *E. coli* 25922 (i.e., Pen-BR and Pen-RRR: 32 μM; CapM2: 64 μM; CECP1: 8 μM). As shown in Figure 1, Pen-BR and Pen-RRR peptides exhibited gradual bactericidal effect overtime. However, Pen-RRR displayed a faster killing rate than Pen-BR with full reduction in viability after 3.5-h treatment, while Pen-BR required overnight incubation (data not shown). CECP1 exhibited similar bactericidal kinetics as Pen-BR (Figure 1) and required overnight treatment for complete killing (data not shown). CapM2 showed the most rapid killing activity among the four peptides examined with complete killing of *E. coli* in 1.5 h (Figure 1). CapM2 is known as a potent membrane permeabilizing antimicrobial peptide (Okuda et al., 2009). Therefore, the fast killing mediated by CapM2 is expected. In addition, the different kinetics observed between CapM2 and HEXIM1 BR peptides might suggest that different antimicrobial mechanisms were utilized by CapM2 and BR peptides.

### Inhibition of Bacterial Translation by the HEXIM1 BR Peptide

**FIGURE 2:**
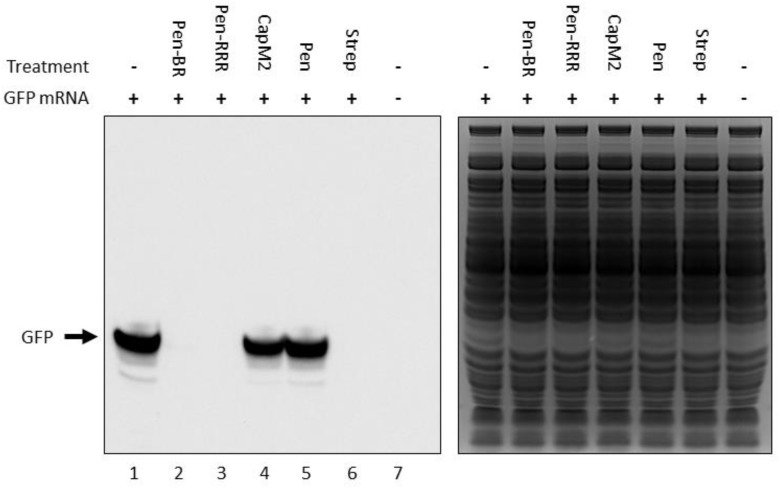
Pen-BR and Pen-RRR inhibit bacterial translation. Rapid translation system (RTS) containing 5 μg GFP mRNA was treated with 100 μM of the indicated peptides and incubated at 30°C for 6 h. RTS with and without GFP mRNA acted as the positive and negative control, respectively, for GFP production, while 10 μM streptomycin (Strep) was used as a positive control for protein translation inhibition. Western blot was performed to determine GFP protein levels after peptide treatment (left panel). SDS–PAGE gel after the transfer step was stained with Coomassie blue dye for loading control (right panel).

In our previous study, we found the HEXIM1 BR peptide was located within the nucleolus and led to degradation of several nucleolar proteins (Neo et al., 2016). Since nucleolus is an important organelle for ribosome biogenesis, we suspect the HEXIM1 peptide may interfere with protein translation. Although bacteria do not contain nucleoli, it is still possible that the HEXIM1 peptides may target translational machinery which is conserved between prokaryotes and eukaryotes. To determine if the BR peptides utilized a similar killing mechanism, we tested the effect of Pen-BR and Pen-RRR on protein translation using an *in vitro* rapid translation system (RTS) kit. The RTS kit contains *E. coli* lysate, in which the protein is synthesized through bacterial translational process, and a GFP expression vector. The RTS kit is designed to perform both transcription and translation in a single reaction. As we only wished to determine the effects of our peptides on bacterial translation, we first synthesized the GFP mRNA using an *in vitro* transcription kit, added the mRNA in the RTS kit, and then evaluated the protein levels of GFP with or without Pen-BR/Pen-RRR peptides by Western blot. Compared to the controls (i.e., without treatment and treated with Pen peptide; Figure 2, lanes 1 and 5), synthesis of GFP protein was almost completely inhibited when Pen-BR or Pen-RRR was included in the reaction (Figure 2, lanes 2 and 3). An antibiotic, streptomycin, which interfered with bacterial translation, acted as a positive control. Treatment with streptomycin resulted in significant inhibition of GFP synthesis (Figure 2, lane 6). We also included another negative control, CapM2, in the experiment. CapM2, known as a membrane permeabilizing antimicrobial peptide, only exhibited limited effects on bacterial translation as expected (Figure 2, lane 4). Taken together, the results suggest that the Pen-BR peptide functions as a novel inhibitor of bacterial translation.

### Cytotoxic Activity of the HEXIM1 BR Peptide on Human Keratinocytes

**FIGURE 3:**
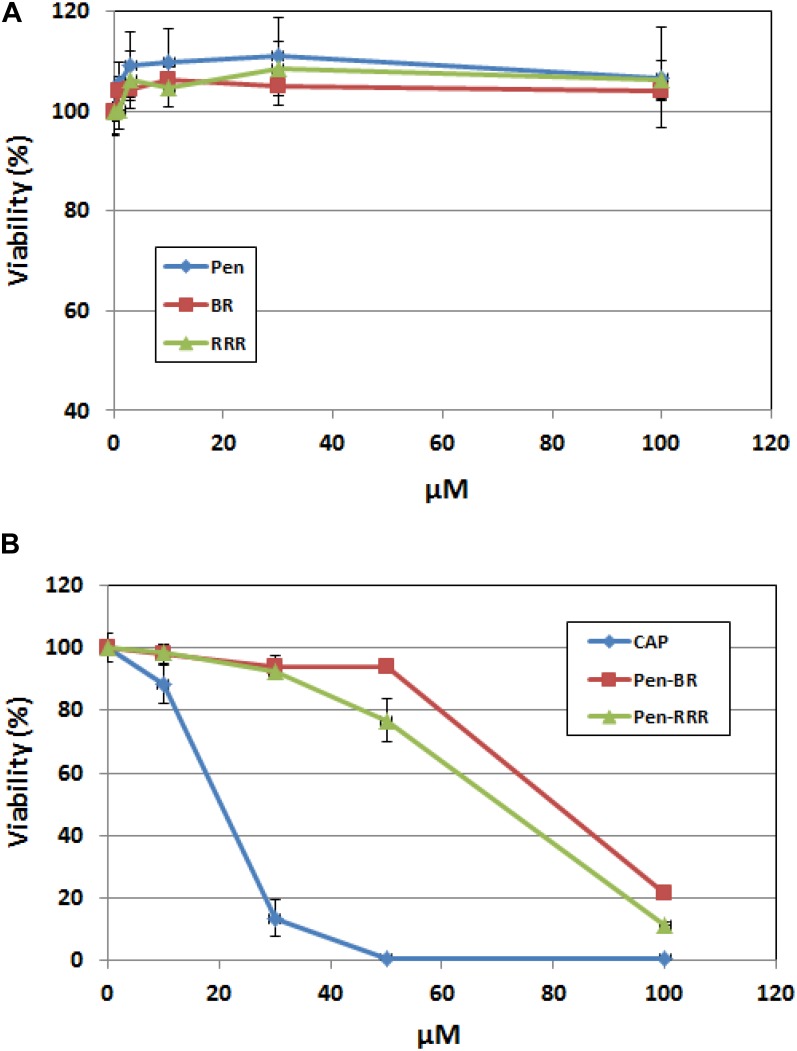
Pen-BR and Pen-RRR exhibit low cytotoxicity against HaCat cells. **(A)** HaCat cells were treated with Pen, BR, and RRR peptides at the serially diluted concentrations (1 to 100 μM) and incubated overnight. **(B)** HaCat cells were treated with CapM2 (Cap), Pen-BR, and Pen-RRR peptides at the indicated concentrations (10, 30, 50, and 100 μM) and incubated overnight. Cell viability was measured by the CellTiter-Glo assay as described in Materials and Methods.

To access the safety profile of the Pen-BR peptide, we next examined the peptide’s effects on mammalian epithelial cells using human keratinocytes. Cell viability of the peptide-treated cells was determined using the CellTiter Glo assay, which was based on quantitation of ATP amounts in the cells. Up to 100 μM, none of the Pen, BR, or RRR12 peptides exhibited any cytotoxic activities on a human keratinocyte cell line, HaCaT (Figure 3A). Pen-BR and Pen-RRR exerted toxic activity against HaCaT cells only at the highest concentration examined (100 μM). Under lower concentrations, Pen-BR did not cause any significant effects, while Pen-RRR exhibited little inhibition on HaCaT cells at 50 μM (about 20% inhibition; Figure 3B). We next checked the effect of CapM2 on HaCaT cells. CapM2 was chosen because both CECP1 and CapM2 were membrane permeabilizing antimicrobial peptides and CapM2 exhibited stronger antimicrobial activities than CECP1 in most bacteria examined here (Table 3). Treatment with 30 μM CapM2 resulted in a 90% decrease in proliferation of HaCaT cells and complete killing was observed when higher concentrations of CapM2 were used (Figure 3B). At 30 μM, both Pen-BR and Pen-RRR killed all the bacterial strains examined in our study (Table 3), while only exhibiting little or no cytotoxicity against HaCat cells (Figure 3B). Such safety profile was not detected in CapM2 as CapM2 kills bacteria and HaCaT cells at the same concentrations (Table 3 and Figure 3B).

We next measured the viability of the peptide-treated bacteria (*E. coli 25922*) using BacTiter-Glo assay which quantified the bacterial ATP. Comparable inhibitory effects were detected among Pen-BR, Pen-RRR, and CapM2 at 3 μM or higher (Supplementary Figure S2). Since CapM2 exhibits stronger cytotoxic effects against HaCat cells than Pen-BR and Pen-RRR, Pen-BR and Pen-RRR still represent as safer treatment options against bacteria.

## Discussion

In this study, we demonstrated the antimicrobial activity of the Pen-BR peptides against antibiotic resistant bacteria. The four selected antibiotic resistant bacteria examined in our study are all listed as “superbugs” by WHO with critical and high priority (World Health Organization [WHO], 2017). No antimicrobial activity was observed when bacteria were treated with HEXIM1 BR peptide alone, suggesting that the BR peptide might not be able to disrupt bacterial plasma membrane or cell wall by itself. The BR-RRR12 mutant peptide was suggested to have weak cell penetrating ability due to the sequence similarity to a cell penetrating peptide (Arg)_9_ (QLGRRRHRRRPSRRRRHW compared to RRRRRRRRR) (Herce et al., 2009). Thus, limited inhibitory effects on the bacteria treated with BR-RRR12 was detected (Table 3). When fused with a cell penetrating peptide, Pen, both Pen-BR and Pen-RRR demonstrated stronger killing ability than two known antimicrobial peptides, CapM2 and CECP1 (Table 3). Importantly, negligible effects on the viability of HaCaT cells were observed when the bactericidal dosages of Pen-BR and Pen-RRR were used (Table 3 and Figure 3). Such safety feature was not detected in CapM2 peptide as CapM2 exhibited similar cytotoxic effects against bacteria and HaCaT cells at the same dosage (Table 3 and Figure 3), indicating that Pen-BR and Pen-RRR could be the better therapeutic options against antibiotic resistant bacteria. Since Pen-BR and Pen-RRR exhibit broad spectrum antimicrobial activities against different bacteria, it would be interesting to further examine their effects on biofilms (de la Fuente-Nunez et al., 2016). Better therapeutic efficacy against biofilms may be achieved in combination with Pen-BR and other existing antibiotics (Reffuveille et al., 2014). Another potential application of the peptides is for the treatment of skin and wound infection, as the human immortalized keratinocyte HaCaT cell model indicated non-toxicity at bactericidal dosages. *S. aureus* and *P. aeruginosa* are the most frequent cause of skin and wound infections and increasing bacterial resistance to conventional antibiotics are becoming a crucial clinical issue. In addition to skin and soft tissue infections, *S. aureus* and *P. aeruginosa* also prolong the inflammatory phase of wound healing (Pfalzgraff et al., 2018). Hence, the topical application of broad-spectrum antimicrobial peptides like Pen-BR and Pen-RRR could facilitate healing by treating the chronic underlying infection.

Our results presented here suggest that translational blockage may be an important mechanism utilized by the HEXIM1 BR peptide in bacterial killing. However, it is possible that the BR peptide may not only affect bacterial translation since it is common that antimicrobial peptides may disrupt multiple biological processes of bacteria (Otvos, 2005). In our previous study, we revealed the multiple mechanisms utilized by the HEXIM1 BR peptide in mammalian cell killing (Neo et al., 2016). Treatment with the BR peptide causes the depolarization of mitochondrial membrane potential, which may reduce or inhibit the production of ATP, resulting in rapid death in mammalian cells (Neo et al., 2016). Furthermore, disorganization of the nucleoli in the treated cells is also detected (Neo et al., 2016). Since the nucleolus is important for ribosome assembly and protein translation, we hypothesize that the BR peptide may affect the translation of mammalian cells and bacteria. Moreover, in another study, a mass spectrometry-based approach identifies several proteins required for DNA replication/repair, cell cycle regulation, and mRNA transcription present in the nucleolus, suggesting the involvement of the nucleolus in other essential biological processes (Andersen et al., 2005). Thus, this may also help to explain why nucleolar disorganization caused by the BR peptide can trigger rapid and potent killing in mammalian cells. We believe that the BR peptide does not use the identical mechanisms to kill bacteria since bacteria do not have mitochondria and nucleoli. In addition, as shown in our earlier study, when conjugated with a cell penetrating peptide, the internalized BR peptide kills mammalian cells in minutes (Neo et al., 2016) while it takes hours for bacterial killing (Figure 1). Since cell death caused by translational inhibition would take hours to initiate, inhibition of translation should not be the main cytotoxic mechanism used by the BR peptide in mammalian cells.

CapM2 and Pen-BR exhibited different kinetics in bacterial killing. CapM2, an optimized membrane-penetrating peptide, killed bacteria completely in 1.5 h (Figure 1). Overnight incubation was required for Pen-BR to eliminate bacteria totally (Figure 1) (data not shown). As Pen did not show any toxic effects on bacteria and BR peptide was shown to block bacterial translation, longer killing time was expected for bactericidal activity of the BR peptide. Pen-RRR displayed faster killing than Pen-BR. Since the RRR12 mutant contained cell penetrating ability (data not shown) (Neo et al., 2016), it could be possible that Pen-RRR internalized into bacteria much faster than Pen-BR and then interfered with bacterial protein synthesis. Another possibility is that the limited cell penetrating ability of RRR12 peptide might also disrupt the membrane structure of bacteria, leading to faster killing.

The HEXIM1 BR peptide can not enter mammalian cells or bacteria by itself and requires a guiding peptide (such as a cell penetrating peptide) for directed therapy. Such a unique feature of the BR peptide can prevent non-specific toxicity to mammalian cells when treated with the BR fusion peptide. On the other hand, it also illustrates the importance of cell penetrating peptides in drug delivery and targeted therapy. Up-to-date, more than 1700 cell penetrating peptides have been reported in a database of cell penetrating peptides (Gautam et al., 2012) and some of the peptides have been shown to specifically against bacteria (Eckert et al., 2006). In addition, it has been shown that *in silico* methods can be applied to design and optimize cell penetrating peptides to enhance their selectivity (Kumar et al., 2018; Porto et al., 2018).

We noticed that Pen-BR and Pen-RRR also exhibited toxicity against mammalian cells when higher concentrations were used. Besides applying the *in silico* approaches to further improve the specific toxicity of the BR peptide, there are few established strategies that can be used. For example, we can substitute the cell penetrating peptide with a more selective targeting peptide against bacteria (Eckert et al., 2006). Also, alanine substitution approach may be useful to identify residues important for cytotoxicity or bactericidal activity. Irazazabal et al. applied this approach to design a modified antimicrobial peptide with lower cytotoxicity while maintaining its antimicrobial activity (Irazazabal et al., 2016).

Unlike most antibacterial peptides which exert their killing activity by disrupting bacterial membrane, we demonstrated that our HEXIM1 BR peptides block bacterial translation (Figure 2). Proline-rich antimicrobial peptides (PrAMPs) are also known as non-lytic peptides and inhibit protein synthesis of bacteria by targeting ribosomes (Li et al., 2014). The mechanism of translational inhibition by a PrAMP, Onc112, has been revealed by the crystal structure of Onc112 complexed with the *Thermus thermophilus* 70S ribosome. Onc112 peptide was found to bind to both the exit tunnel and the peptidyl transferase center of ribosomes, resulting in inhibition of translation at the initiation step (Roy et al., 2015; Seefeldt et al., 2015). Another PrAMP, Api137, also blocked protein synthesis but inhibited bacterial translation at the termination stage (Florin et al., 2017). Since the sequence of HEXIM1 BR peptides is quite different from that of PrAMPs, it is likely that different mechanism of action may be utilized by the BR peptide. Besides having an impact on NPM, treatment with BR peptide in mammalian cells also led to decreases in protein levels of certain ribosomal subunits (data not shown) (Neo et al., 2016). Future investigation is required to elucidate the mechanism of action for BR peptides in bacterial translation. Furthermore, the BR peptide may have one advantage over PrAMPs. When fused with a cell penetrating peptide, BR peptide can eliminate both Gram-positive and Gram-negative bacteria (Table 3), while PrAMPs are predominantly active against Gram-negative bacteria (Scocchi et al., 2011; Li et al., 2014).

In conclusion, we examined the antimicrobial properties of the HEXIM1 BR peptide and its derivative, BR-RRR12 and found that both peptides, when fused with a cell penetrating peptide (Pen), exhibited inhibitory activities against a broad spectrum of clinically relevant Gram negative and Gram positive bacteria. At the bactericidal dosages, negligible effects on the viability of human keratinocyte cell line were observed and the peptides were found to blocked bacterial translation when tested on an *E. coli in vitro* expression system. In all, the Pen-BR and Pen-RRR peptides were identified as novel antimicrobial peptides with potent inhibitory activity against drug-resistant “superbugs.”

## Author Contributions

PH and HO performed the experiments and drafted the manuscript. JT provided the materials and revised the manuscript. S-HC and DO designed the experiments, supervised the research work, and revised the manuscript.

## Conflict of Interest Statement

The authors declare that the research was conducted in the absence of any commercial or financial relationships that could be construed as a potential conflict of interest.
