# Supplementary material for: HEXIM1 Peptide Exhibits Antimicrobial Activity Against Antibiotic Resistant Bacteria Through Guidance of Cell Penetrating Peptide
Source: Front Microbiol. 2019 Feb 8;10:203. doi: 10.3389/fmicb.2019.00203 (PMC6376162; doi:10.3389/fmicb.2019.00203)

**Supplementary Figure Legends**

**Supplementary Figure S1: Schematic overview of Pen-HEXIM1 BR fusion peptides.** Pen, a cell penetrating peptide, is placed at the N-terminus and fused to an antimicrobial peptide (i.e. HEXIM1 BR or RRR12 peptides) through a Gly-Gly (i.e. GG) linker to form the Pen-BR and Pen-RRR peptides, respectively.

**Supplementary Figure S2**: **Pen-BR and Pen-RRR inhibit the generation of ATP in bacteria.** *Escherichia coli* ATCC 25922 cells grown to mid-log phase was diluted in PBS and added to the wells containing peptides to achieve a final concentration of 1 x 10^5^ cells.mL^-1^. Peptides used include Pen, Pen-BR, Pen-RRR, BR, RRR12 and CapM2, with final concentrations at 0.1, 0.3, 1, 3 and 10 µM. The incubation was performed on 96-well black plates (Grenier Bio-One) for 20 hours at room temperature. BacTiter-Glo assay (Promega) was then used according to manufacturer’s protocol to measure the cell viability.


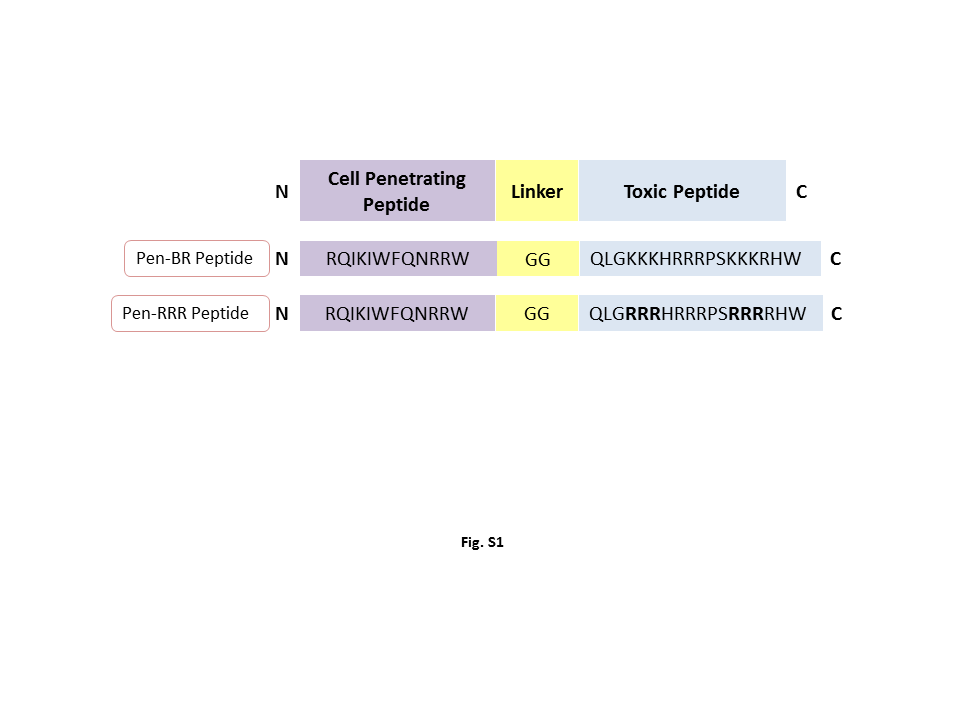


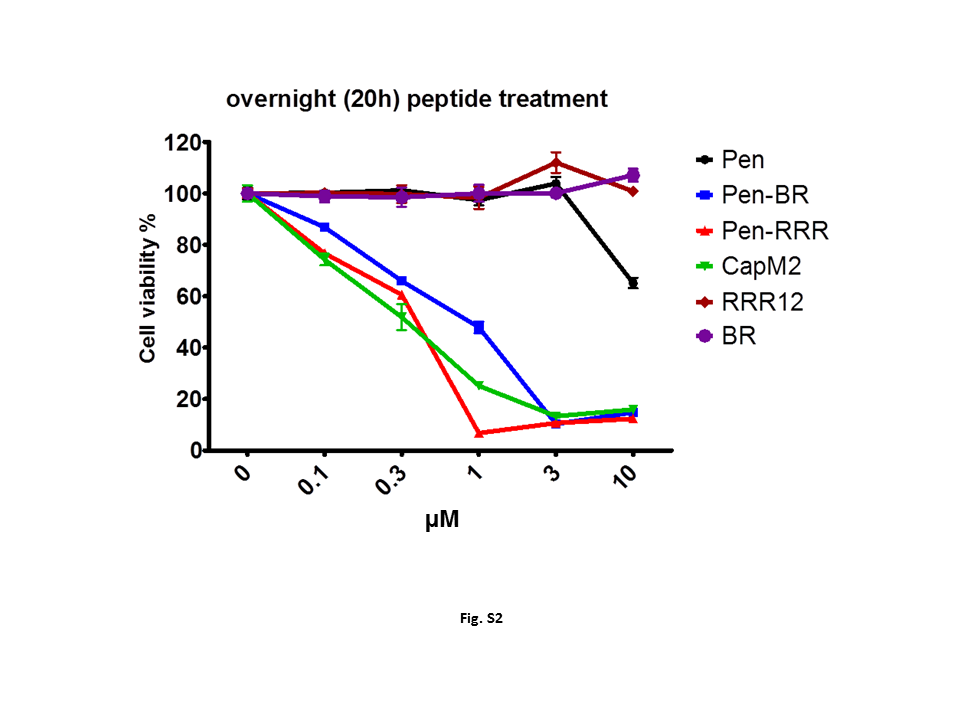

Supplement: Supplementary file 1 [file Data_Sheet_1.docx]
